# Supplementary material for: Development of a computer-aided design software for the quantitative evaluation of aesthetic damage
Source: PLoS One. 2019 Dec 18;14(12):e0226322. doi: 10.1371/journal.pone.0226322 (PMC6919621; doi:10.1371/journal.pone.0226322)
Supplement: S1 Code site — 1. (ZIP) [file pone.0226322.s001.zip › [EESC_jr][Projeto]Relatorio_07_21/vendors/select2/docs/_includes/options/selections/placeholder.html]

## How can I have Select2 display a placeholder?

Select2 supports displaying a placeholder by default using the `placeholder` option. This can be either a data object matching the placeholder option, or a string to display as the placeholder if you are using a blank placeholder option.

{% highlight js linenos %}
$('select').select2({
placeholder: 'Select an option'
});
{% endhighlight %}

### My first option is being displayed instead of my placeholder

This usually means that you do not have a blank `<option></option>` as the first option in your `<select>`.

Note that this does not apply to multiple selects, as the browser does not select the first option by default when multiple selections can be made.

### I am using AJAX, can I still show a placeholder?

Yes, Select2 supports placeholders for all configurations. You will still need to add in the placeholder option if you are using a single select.

### Can I use an option without a blank value as my placeholder?

The `placeholder` option allows you to pass in a data object instead of just a string if you need more flexibility. The `id` of the data object should match the `value` of the placeholder option.

{% highlight js linenos %}
$('select').select2({
placeholder: {
id: '-1', // the value of the option
text: 'Select an option'
}
});
{% endhighlight %}

### Can I change how the placeholder looks?

When using Select2 **when only a single selection can be made**, the placeholder option will be passed through the standard templating methods, including the `templateSelection` option, so you are able to change how it is displayed.

{% highlight js linenos %}
$('select').select2({
templateResult: function (data) {
if (data.id === '') { // adjust for custom placeholder values
return 'Custom styled placeholder text';
}
return data.text;
}
});
{% endhighlight %}

**When multiple selections are allowed**, the placeholder will be displayed using the `placeholder` attribute on the search box. You can cusotmize the display of this placholder using CSS, as explained in the following Stack Overflow answer: Change an input's HTML5 placeholder color with CSS

### My placeholders aren't being displayed in Internet Explorer

Select2 uses the native `placeholder` attribute on input boxes for the multiple select, and that attribute is not supported in older versions of Internet Explorer. You need to include Placeholders.js on your page, or use the full build, in order to add `placeholder` attribute support to input boxes.
